# Comparison of Trial Participants and Open Access Users of a Web-Based Physical Activity Intervention Regarding Adherence, Attrition, and Repeated Participation

**DOI:** 10.2196/jmir.1361

**Published:** 2010-02-10

**Authors:** Miriam Wanner, Eva Martin-Diener, Georg Bauer, Charlotte Braun-Fahrländer, Brian W Martin

**Affiliations:** ^4^Division of Public and Organizational HealthUniversity and ETH ZurichZurichSwitzerland; ^3^Institute of Social and Preventive MedicineUniversity of ZurichZurichSwitzerland; ^2^Swiss Tropical and Public Health InstituteUniversity of BaselBaselSwitzerland; ^1^Swiss Federal Institute of Sport MagglingenMagglingenSwitzerland

**Keywords:** Internet, individually tailored intervention, user characteristics, time trends

## Abstract

**Background:**

Web-based interventions are popular for promoting healthy lifestyles such as physical activity. However, little is known about user characteristics, adherence, attrition, and predictors of repeated participation on open access physical activity websites.

**Objective:**

The focus of this study was Active-online, a Web-based individually tailored physical activity intervention. The aims were (1) to assess and compare user characteristics and adherence to the website (a) in the open access context over time from 2003 to 2009, and (b) between trial participants and open access users; and (2) to analyze attrition and predictors of repeated use among participants in a randomized controlled trial compared with registered open access users.

**Methods:**

Data routinely recorded in the Active-online user database were used. Adherence was defined as: the number of pages viewed, the proportion of visits during which a tailored module was begun, the proportion of visits during which tailored feedback was received, and the time spent in the tailored modules. Adherence was analyzed according to six one-year periods (2003-2009) and according to the context (trial or open access) based on first visits and longest visits. Attrition and predictors of repeated participation were compared between trial participants and open access users.

**Results:**

The number of recorded visits per year on Active-online decreased from 42,626 in 2003-2004 to 8343 in 2008-2009 (each of six one-year time periods ran from April 23 to April 22 of the following year). The mean age of users was between 38.4 and 43.1 years in all time periods and both contexts. The proportion of women increased from 49.5% in 2003-2004 to 61.3% in 2008-2009 (*P*< .001). There were differences but no consistent time trends in adherence to Active-online. The mean age of trial participants was 43.1 years, and 74.9% were women. Comparing contexts, adherence was highest for registered open access users. For open access users, adherence was similar during the first and the longest visits; for trial participants, adherence was lower during the first visits and higher during the longest visits. Of registered open access users and trial participants, 25.8% and 67.3% respectively visited Active-online repeatedly (*P*< .001). Predictors of repeated use were male sex (odds ratio [OR] = 1.2, 95% confidence interval [CI] = 1.04-1.38) and increasing age category in registered open access users, and age 46-60 versus < 30 years (OR = 3.04, 95% CI = 1.25-7.38) and Swiss nationality (OR_*nonSwiss*_= 0.64, 95% CI = 0.41-1.00) in trial participants. Despite reminder emails, attrition was much higher in registered open access users compared with trial participants, with a median lifetime website usage of 0 days in open access users and 290 days in trial participants.

**Conclusions:**

Adherence, patterns of use, attrition, and repeated participation differed between trial participants and open access users. Reminder emails to encourage repeated participation were effective for trial participants but not for registered open access users. These issues are important when interpreting results of randomized controlled effectiveness trials.

## Introduction

In recent years,Web-based interventions targeting health issues such as nutrition [1], smoking [2], physical activity [3,4], or multiple health behaviors [5] have become popular. These interventions have several advantages, such as interactive designs, the possibility of tailoring information to individual users, the potential to reach large audiences at relatively low costs, and the ease with which users can get involved, that is, people can use the intervention at home.

To maximize effectiveness, it is important for developers of such interventions to know more about user characteristics, adherence (the extent to which individuals use the content of the Internet intervention) [6], nonusage attrition (whether individuals discontinue use of an Internet intervention) [7], and predictors of repeated Internet intervention use. Studies reporting these issues have done so mostly in the context of randomized controlled trials (RCT) [8-13] or in other controlled study settings [14]. However, the use of the intervention in a trial context may not reflect the use of the intervention in an open access context. This is an important issue, as program effectiveness is likely to depend on adherence to and use of websites. If adherence is higher among trial participants (eg, due to higher motivation among trial participants and the efforts of the study staff to increase adherence), effectiveness may be overestimated; if adherence is lower among trial participants (eg, due to a higher burden of additional data assessments), effectiveness may be underestimated.

Few studies have described use and users of open access websites in the domain of smoking [15,16], mental health [17,18], and drinking [19]. Even fewer studies have done so in the domain of physical activity. To our knowledge, there is only one study that has described rates and determinants of repeated participation in an open access Web-based program aimed at healthy lifestyles that has emphasized healthy body weight and physical activity [20].

In Switzerland, a Web-based tailored physical activity intervention (Active-online) [21] developed between 1999 and 2003, has been freely available as an open access program since 2003. Continuous data collection pertaining to website visitors provides an opportunity to analyze user characteristics and patterns of intervention use and adherence over time.The effectiveness of the intervention was assessed in a Web-based RCT in 2006-2007 [22] after the study design had been tested in a feasibility study in 2003 [23].

The aims of the present study were: (1) to assess and compare user characteristics and adherence to the website (a) in an open access context over time from 2003 to 2009, and (b) between participants in an RCT and open access users (all open access users and the subgroup of registered open access users only), and (2) to analyze attrition and potential predictors of repeated use of the website in trial participants compared with registered open access users.

## Methods

### Intervention Program

**Figure 1:**
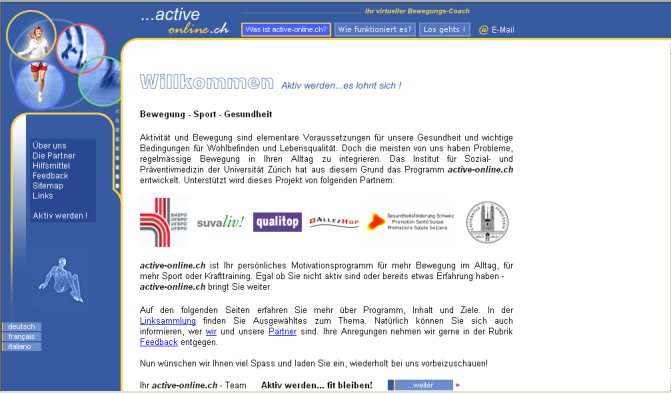
Screenshot of the welcoming page of Active-online

**Figure 2:**
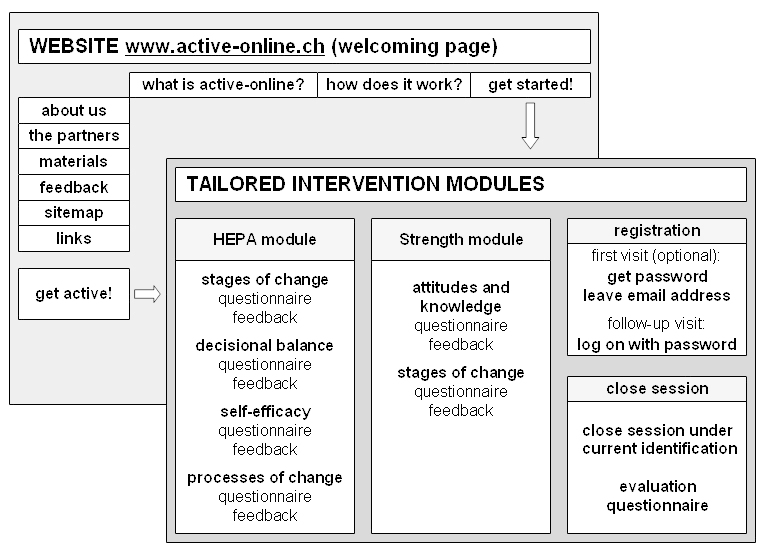
Structure of Active-online and the tailored intervention modules

Active-online is an individually tailored program to promote physical activity targeting adults aged 30 to 60 years. Active-online is freely available on the Internet in the three main languages of Switzerland: German, French, and Italian. At the start of the program, users find a language selection page followed by a welcoming page that explains the program and provides additional information and motivational material (see Figure 1 and Figure 2). At the beginning of the tailored intervention, a new window opens where two modules are offered: one module on everyday health-enhancing physical activity (HEPA) and endurance training (the HEPA module), and one module on strength and flexibility training (the strength module) (Figure 2). Visits are recorded in the database as soon as a new window is opened (after the welcoming page) where one of the two tailored modules can be selected.

Users may visit Active-online without registering, or they may register. Registration is very brief and involves leaving an email address. Registered users receive a password which allows them to revisit the website and follow changes in their physical activity behavior. Registered users receive reminder emails that contain a link to revisit the website after 2, 4, and 7 months.

The HEPA module offers a maximum of four individually tailored feedbacks based on the transtheoretical model of behavior change [24]. Stages of change are assessed according to a seven-stage concept focusing on current behavior (moderate- and vigorous-intensity activities) as well as on intention to change [25]. The module on strength and flexibility training offers a maximum of two tailored feedbacks. Questionnaires preceding each feedback include between 5 and 23 questions and can be completed in a few minutes each. It is unlikely that visitors complete all parts of both tailored modules during one visit, thus repeated visits are encouraged (with reminder emails to registered users). Moreover, repeated visitors who answer the questions obtain individually tailored feedback on changes since their last visit.

### Randomized Controlled Effectiveness Trial

The RCT is described in detail elsewhere [22]. Briefly, participants were recruited in 2006 by advertisements in magazines, newspapers, and on websites. The advertisements asked for volunteers to participate in a Web-based physical activity study. After completing an online baseline questionnaire with items on demographics, general health, and physical activity behavior, participants assigned to the intervention group were forwarded to the open access program, Active-online, and directed to use the intervention. (Participants assigned to the control group were forwarded to a simple nontailored website that contained general information on physical activity and health.) To replicate the conditions of open access use, participants assigned to the intervention group had access to the general instructions on the website; they did not receive additional instructions in how to use the intervention. All contacts were by email. Trial participants received reminder emails to revisit the Active-online website at 9, 10, and 11 months after baseline. (Participants assigned to the control group received no reminder emails.) Follow-up assessments as part of the requirements of the RCT took place at 6 weeks, 6 months, and 13 months. While registration on Active-online was not compulsory for open access users, trial participants were automatically registered in order to analyze their intervention use.

### Time Periods of Intervention Use, Data Collection, and Variables Included in the Analyses

In April 2003, Active-online was officially launched with a promotional event. Data from the open access period were included for six one-year time periods through April 2009. Each one-year time period started on April 23 and ended on April 22 of the following year. Data from the effectiveness trial were included from May 1, 2006, through September 30, 2007.

The total number of visits on Active-online (including visits on the welcoming page) was available from the Internet provider. However, the absolute numbers were difficult to interpret because these depended on the software used to assess them and whether visits by web crawlers could be identified, for example. For both open access users and trial participants, visits on Active-online were captured in the Active-online user database as soon as the new browser window for the selection of one of the two tailored modules was opened (see Figure 2), whereas visits that did not go beyond the welcoming page were not recorded.

For each visit that was captured in the Active-online user database, starting time and date, finishing time and date, number of pages viewed, and time spent within the tailored modules were recorded in addition to responses to the questionnaires preceding each tailored feedback.

**Figure 3:**
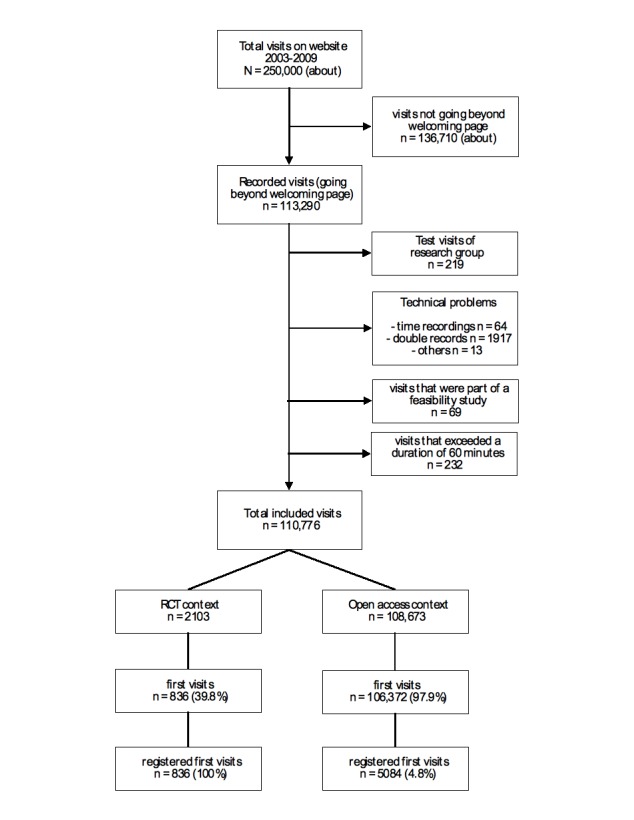
Inclusion and exclusion of records used in the analyses

During the study period, between April 23, 2003, and April 22, 2009, more than 250,000 visits were counted on Active-online (including visits not going beyond the welcoming page and excluding visits by web crawlers). For the present study, only those visits that went beyond the welcoming page and were recorded in the Active-online user database during the study period were included. These numbered 113,290. Figure 3 shows the inclusion and exclusion of visits that served as the basis for the analyses in this study. We excluded 219 visits that were obvious test visits of the research group and another 64 visits because the start times and end times did not correspond. Furthermore, 232 visits were excluded because they exceeded 60 minutes as we assumed these visits were not properly terminated. Users who received all tailored feedbacks, read them online, and used the option to download further materials could easily have spent half an hour or more on Active-online.The rationale to use a cutoff of 60 minutes was to allow for potential distractions during a visit and to make sure that no serious visits were excluded. Another 13 visits were excluded due to other recording problems, and 1917 records were deleted because they represented double visits of the same session. Finally, 69 records of individuals who had participated in the feasibility study [23] were dropped. Thus, 110,776 visits were included, of which 107,208 (96.8%) were recorded as first visits.

Of the 110,776 visits, 108,673 were recorded in the open access context and 2103 during the RCT. For the analysis of time trends, open access visits were stratified according to the six one-year periods between 2003 and 2009. The number of recorded and first visits and the proportion of first visits resulting in registration are displayed in Table 1 for each time period and for the RCT. Of all open access visits, 2.1% were recorded as repeated visits. The corresponding proportion was 60.2% during the RCT.

**Table 1:** Recorded website visits in the open access context (according to the six time periods from 2003-2009) and during the RCT

Open Access Program Use	Number of Visits Recorded in Database	Number of First Visits (% of Recorded Visits)	Number of First Visits Resulting in Registration (% of First Visits)
**Time period (from April 23 to April 22 of the following year) **			
2003-2004	42,626	41,699 (97.8%)	2263 (5.4%)
2004-2005	25,392	25,026 (98.6%)	784 (3.1%)
2005-2006	12,776	12,517 (98.0%)	592 (4.7%)
2006-2007	9847	9539 (96.9%)	610 (6.4%)
2007-2008	9689	9451 (97.5%)	513 (5.4%)
2008-2009	8343	8140 (97.6%)	322 (4.0%)
Total (2003-2009)	108,673	106,372 (97.9%)	5084 (4.8%)
** RCT^a^ (May 1, 2006 to September 30, 2007)**	**2103 **	**836 (39.8%) **	**836 (100%) **

^a^Trial participants were automatically registered within Active-online.

The proportion of women among Active-online users and the mean age of visitors were included in the analyses as demographic variables. The main measure of physical activity was the proportion meeting the current Swiss recommendations for health-enhancing physical activity (HEPA): 30 minutes or more of moderate intensity activities on 5 or more days per week or 20 minutes or more of vigorous intensity activities on 3 or more days per week [26]. Additional potential predictors of repeated participation were available for trial participants only. These were smoking, BMI, education, and nationality. For the comparison of repeated participation between open access users and trial participants, only registered open access users were included (n = 5084, 4.8% of all open access users) because repeated visits could only be tracked for participants who had registered.

Adherence, defined as the extent to which individuals experienced the content of the website [6], is reported as the number of pages viewed on Active-online, the proportion of visits that resulted in starting a tailored module, the proportion of visits when at least 3 minutes were spent in a tailored module (assuming that a minimum of 3 minutes is required to get involved with the intervention), the proportion of visits when at least one tailored feedback (HEPA or strength module, see Figure 2) was received, and the time spent in the tailored modules. These measures of adherence are commonly used to describe the extent to which individuals use the material on Web interventions [6,13]. Analysis of adherence was based on first visit to compare open access dissemination of the intervention across time periods, and on first visit and longest visit to compare adherence of open access users with adherence of trial participants. During open access dissemination, most visits were first visits so that analyzing by longest visit yielded almost identical results.

Attrition describes the phenomenon that participants stop using the intervention [7]. Nonusage attrition refers to participants not returning to the intervention for repeated visits [7]. We also report attrition in terms of the duration of a single visit. In this sense, attrition refers to users who discontinue their visit at a specific point in time versus those who continue their visit beyond that point.

Comparisons are reported between trial participants (all of whom were registered, according to the study design), open access users (including both registered and unregistered open access users), and the subgroup of open access users who had registered and received a password to revisit Active-online.

### Statistical Analyses

Demographic variables were compared between open access users and trial participants using *t*-tests and chi-square tests. Continuous variables measuring the use of Active-online (number of pages viewed and time spent in the tailored modules) were positively skewed. For these variables, therefore, the median and the interquartile range (IQR) are reported. The Wilcoxon-Mann-Whitney test and the chi-square test were used to compare use of Active-online between trial participants and open access users. Assuming that differences between time periods followed a time trend rather than a random pattern, a nonparametric test for trend across ordered groups developed by Cuzick [27] was performed for continuous variables, and a chi-square test for trend was performed for categorical variables to assess potential time trends across the six time periods. Logistic regression was used to assess potential predictors of repeated use of the website.

Nonusage attrition curves were based on the proportion of visitors still using the website up to a specific number of weeks or months after the first visit versus those who had stopped using it. The date of each user's last visit was designated as the date when program usage ended. The nonusage attrition curves are presented over 18 months (considered a suitable timeframe for trial participation) and over 12 weeks (for comparison with other published attrition curves). Similarly, attrition curves based on the duration of single visits (first visits and longest visits) are presented, which correspond to the proportion of visitors who had continued to use the intervention within a single session versus those who had ended the session after a specific number of minutes. Duration was defined as the time spent in the tailored modules as recorded in the user database; therefore, individuals that did not enter a tailored module have been assigned a duration of zero. STATA 9.2 (STATACorp LP, College Station, TX, USA) was used for the analyses.

## Results

### User Characteristics

The yearly number of open access visits recorded in the Active-online database decreased from 42,626 in 2003-2004 to 8343 in 2008-2009. In the open access context, the proportion of women using Active-online increased from 49.5% in 2003-2004 to 61.3% in 2008-2009 (*P *[for trend] < .001). The mean age of open access users was between 38.4 years (95% confidence interval [CI] 38.0-38.8) in 2008-2009 and 40.4 years (95% CI 40.1-40.7) in 2005-2006 and 2006-2007. The proportion of open access users meeting the HEPA recommendations was between 39.9% in 2004-2005 and 42.6% in 2005-2006 (*P *[for trend] = .015).

Among open access Active-online users, 55.1% were women, while 74.9% of trial participants were women (*P *< .001). The mean age of open access users was 39.1 years (95% CI 39.0-39.2) compared with a mean age of trial participants of 43.1 years (95% CI 42.2-44.0) (*P *< .001). The proportion of individuals meeting the current Swiss HEPA recommendations did not differ significantly between open access users (40.9%) and trial participants (44.5%, *P*= .27).

### Adherence to and Use of the Intervention

There were differences, but no consistent trends over time, in adherence to Active-online among open access users (based on the analysis of first visits). In general, use of the intervention among open access users was higher in 2003-2004, 2005-2006, and in 2006-2007, but lower in 2004-2005 and after 2007 (Table 2). Between 2003 and 2009, open access users who started a tailored module spent an average of 7.5 minutes in the program, with a median duration of 4.2 minutes. The subgroup of registered open access users who started a tailored module spent an average of 17.7 minutes in the program, with a median duration of 15.0 minutes.

For first visits, adherence to Active-online was highest for registered open access users (Table 2). Compared with all open access users, adherence was lower among trial participants. Trial participants visited fewer pages, and the proportion that started a tailored module, that spent at least 3 minutes in the modules, and that received at least one tailored feedback (HEPA or strength module) was smaller. However, trial participants who started a module tended to stay in the intervention longer.

Analyzing adherence according to longest visit, we found that results remained very similar to the results for first visit among open access users, indicating that the first and longest visit were identical among these users. This was not true for trial participants, however. As was the case for first visits, registered open access users achieved the highest adherence when results were based on longest visit. Trial participants' adherence was considerably higher during the longest visit than during the first visit (indicating that the first visit was not the longest visit) and was higher compared with all open access users.

**Table 2:** Adherence at first visit according to time periods during open access use 2003-2009, and at first and longest visit according to open access context and randomized controlled trial

	Median (IQR) Number of Pages Viewed per Visit	Number of Visits (%) When a Tailored Module Was Started	Number of Visits (%) When ≥ 3 Minutes Spent in the Tailored Modules	Number of Visits (%) When At Least One Tailored Feedback Was Received	Median (IQR) Minutes Spent in Tailored Module per Visit (When a Module Was Started)	Median (IQR) Minutes Spent in Tailored Modules per Visit (if ≥ 3 Minutes Spent in Modules)
**Comparison of time periods^a^ during open access use (based on first visits) **						
2003-2004	16 (9-28)	29,967 (71.9%)	19,349 (46.4%)	24,973 (59.9%)	4.2 (1.8-11.4)	8.4 (4.8-15.6)
2004-2005	11 (4-21)	16,465 (65.8%)	9341 (37.3%)	13,132 (52.5%)	3.6 (1.2-8.4)	7.2 (4.2-13.8)
2005-2006	19 (11-31)	8851 (70.7%)	5593 (44.7%)	7277 (58.1%)	4.2 (1.8-11.4)	9.0 (4.8-15.6)
2006-2007	21 (13-32)	6661 (69.8%)	4154 (43.5%)	5716 (59.9%)	4.2 (1.8-10.8)	8.4 (4.8-15.6)
2007-2008	19 (11-30)	6015 (63.6%)	3728 (39.4%)	5152 (54.5%)	4.2 (1.8-10.2)	8.4 (4.8-14.4)
2008-2009	16 (11-25)	4818 (59.2%)	2639 (32.4%)	4090 (50.3%)	3.0 (1.2-7.8)	6.6 (4.2-11.4)
* P *Value^b^	< .001	< .001	< .001	< .001	< .001	< .001
**Comparison of open access users and RCT participants (based on first visits) **						
Open access: all users	16 (9-27)	72,777 (68.4%)	44,804 (42.1%)	60,340 (56.7%)	4.2 (1.8-10.2)	8.4 (4.8-15)
Open access: registered users only	42 (30-57)	4892 (96.2%)	4643 (91.3%)	4629 (91.1%)	15.0 (8.4-24.0)	15.6 (9.6-24.6)
Trial participants	7 (2-21)	322 (38.5%)	265 (31.7%)	250 (29.9%)	9.0 (3.6-15.6)	10.8 (6.0-16.2)
* P *Value^c^	< .001	< .001	< .001	< .001	< .001	< .001
**Comparison of open access users and RCT participants (based on longest visits) **						
Open access: all users	16 (9-27)	72,943 (68.5%)	44,985 (42.2%)	60,531 (56.8%)	4.2 (1.8-10.2)	8.4 (4.8-15.0)
Open access: registered users only	43 (31-57)	5021 (96.5%)	4789 (92.0%)	4779 (91.8%)	15.6 (9.0-24.6)	16.2 (10.2-25.2)
Trial participants	23 (8-38)	626 (74.4%)	554 (65.8%)	549 (65.2%)	12.0 (5.4-18.6)	13.2 (7.8-20.4)
* P *value^c^	< .001	< .001	< .001	< .001	< .001	< .001

^a^Each one-year time period started on April 23 and ended on April 22.

^b^ *P *values are based onchi-square test for trend (categorical variables over time) and test for trend developed by Cuzick (continuous variables over time).

^c^ *P *values for both the comparison between open access users (all) and trial participants, as well as between registered open access users and trial participants. *P *values are based on chi-square tests (comparisons between open access users and trial participants for categorical variables) and Wilcoxon-Mann-Whitney test (comparisons between open access users and trial participants for continuous variables).

### Attrition

**Figure 4:**
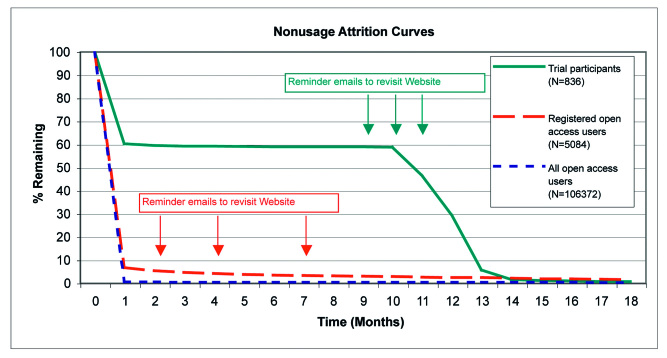
Nonusage attrition curves for open access users and trial participants over 18 months

**Figure 5:**
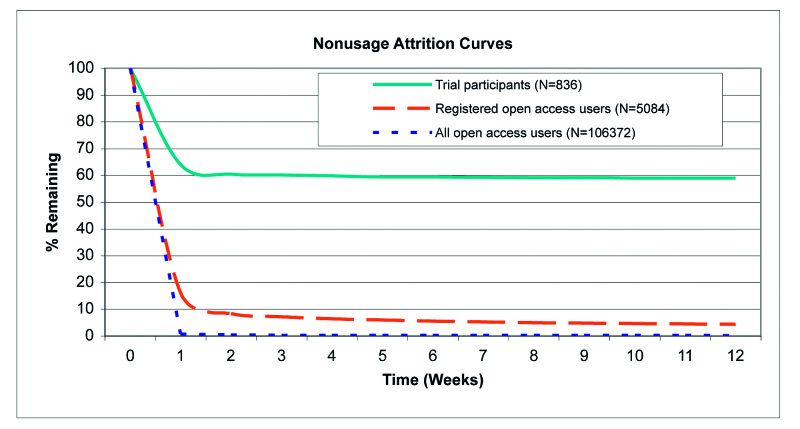
Nonusage attrition curves for open access users and trial participants over 12 weeks

Figure 4 and Figure 5 show nonusage attrition curves for open access users (all open access users and subgroup of registered open access users only) and for trial participants over 18 months and over 12 weeks (for comparison with other published attrition curves, see [7,14]). The median lifetime website usage (time when 50% of users had stopped using the intervention) [13] was 0 days for open access users (all open access users and subgroup of registered open access users only) and 290 days for trial participants. In trial participants, the first two reminder emails after 9 and 10 months resulted in a relatively high proportion of individuals returning to Active-online by clicking on the link in the reminder email; however, fewer individuals returned after the third reminder. Reminder emails sent to registered open access users did not show the same effect: fewer than 6% were still using the website after the first reminder was sent out at two months.

**Figure 6:**
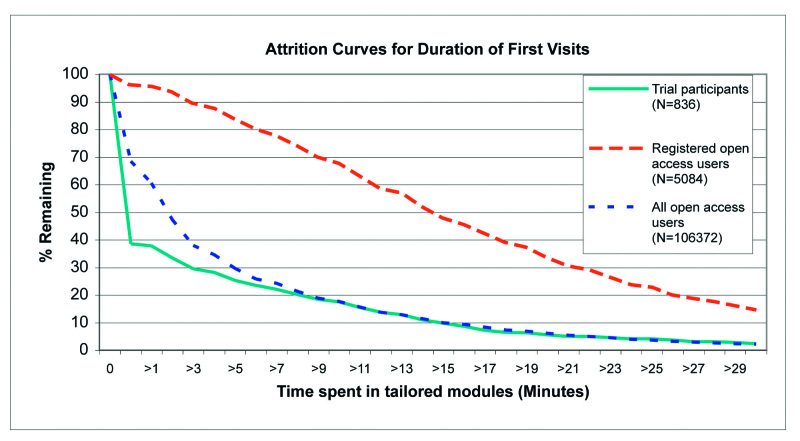
Attrition curves for the duration of the first visit for open access users and trial participants

**Figure 7:**
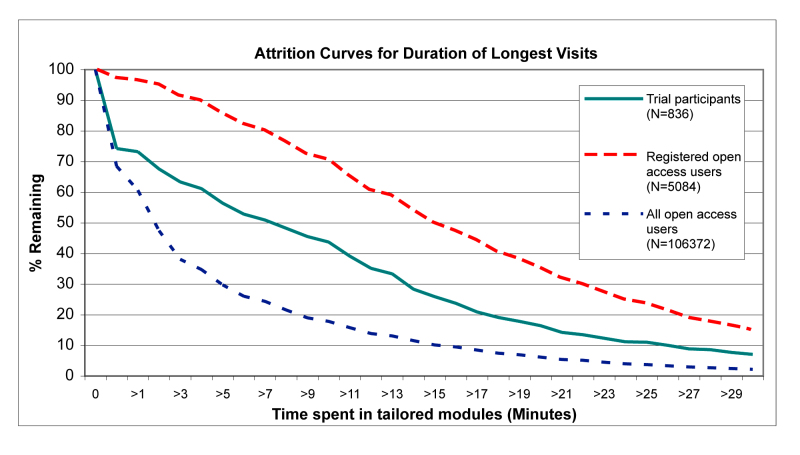
Attrition curves for the duration of the longest visit for open access users and trial participants

Figure 6 and Figure 7 present attrition curves for the time spent in the tailored modules during the first visit and during the longest visit, respectively, for open access users (all open access users and subgroup of registered open access users only) and for trial participants. Registered open access users spent more time in the tailored modules both during their first visit and during their longest visit compared with all open access users and compared with trial participants. The majority of trial participants did not spend much time in the tailored modules during their first visit (Figure 6); however, the proportion spending more time in the tailored intervention was much higher for longest visits (Figure 7). In contrast, the curves are very similar for first and longest visits of open access users (all open access users and subgroup of registered open access users only), indicating that first and longest visits were identical.

### Predictors of Repeated Participation

In total, 1312 (25.8%) of open access users who registered and received a password and 558 (67.3%) of the trial participants returned for a repeated visit (*P *< .001). Table 3 displays potential predictors of repeated participation for registered open access users and for trial participants. Men and older individuals were significantly more likely to visit Active-online repeatedly in the open access context. Among trial participants, only the age group of 46-60 years (compared with < 30 years) was a significant predictor in the adjusted model, while gender did not predict repeated participation. Meeting the HEPA recommendations was not associated with repeated participation in registered open access users. In trial participants, however, there was a nonsignificant tendency for individuals not meeting the HEPA recommendations not to have returned for a repeated visit. Not having Swiss nationality achieved borderline significance as a predictor of lower rates of repeated participation in trial participants. Furthermore, there was a tendency for more highly educated individuals to have returned for a repeated visit; however these associations were not significant. There were no effects for smoking and BMI.

**Table 3:** Predictors of repeated participation for registered open access users and for trial participants

Registered Open Access Users (2003-2009)						Trial Participants			
		N	% Repeated Visits	Unadjusted OR (95% CI)	Adjusted OR^a^(95% CI)	N	% Repeated Visits	Unadjusted OR (95% CI)	Adjusted OR^a^(95% CI)
**Gender**									
	Female	2,886	24.1	1.00	1.00	626	66.3	1.00	1.00
	Male	2,197	28.0	*1.23 *(1.08-1.39)	*1.20 *(1.04-1.38)	210	70.5	1.21 (0.86-1.70)	0.79 (0.39-1.62)
**Age category (years) **									
	< 30	1,270	20.5	1.00	1.00	151	53.6	1.00	1.00
	30-45	2,111	25.9	*1.36 *(1.15-1.60)	*1.37 *(1.13-1.64)	324	65.7	*1.66 *(1.12-2.46)	1.61 (0.71-3.66)
	46-60	1,370	28.2	*1.52 *(1.27-1.82)	*1.48 *(1.21-1.81)	269	75.1	*2.61 *(1.71-3.98)	*3.04 *(1.25-7.38)
	> 60	332	35.8	*2.17 *(1.67-2.82)	*2.26 *(1.68-3.04)	92	72.8	*2.32 *(1.32-4.05)	1.72 (0.57-5.20)
**Met HEPA recommendations **									
	Yes	1,587	24.4	1.00	1.00	347	70.3	1.00	1.00
	No	2,697	25.8	1.07 (0.93-1.24)	1.11 (0.96-1.29)	489	65.2	0.79 (0.59-1.07)	0.76 (0.56-1.03)
**Smoking **		Not available							
	Yes					125	64.8	1.00	1.00
	No					711	67.8	1.14 (0.77-1.70)	1.03 (0.69-1.56)
**BMI **		Not available							
	<= 25					501	67.5	1.00	1.00
	25-30					227	68.7	1.06 (0.76-1.48)	0.93 (0.65-1.32)
	> 30					106	64.2	0.86 (0.56-1.34)	0.79 (0.50-1.25)
**Education **		Not available							
	Compulsory school					29	51.7	1.00	1.00
	Apprenticeship					289	67.8	1.97 (0.91-4.24)	1.73 (0.78-3.81)
	High school					124	63.7	1.64 (0.73-3.70)	1.82 (0.79-4.19)
	Higher professional education, upper vocational school					189	70.9	*2.27 *(1.03-5.03)	1.95 (0.86-4.41)
	University					205	67.8	1.97 (0.90-4.31)	1.74 (0.78-3.91)
**Nationality **		Not available							
	Swiss					737	68.4	1.00	1.00
	Non Swiss					99	59.6	0.68 (0.44-1.05)	*0.64 *(0.41-1.00)

^a^Adjusted for sex, age category, and whether HEPA recommendations were met. Additional adjustment for the other potential predictors in the model (RCT only) did not change the results.

## Discussion

### Principle Results and Comparison with Prior Work

The present study aimed to assess user characteristics, adherence, attrition, and predictors of repeated use in trial participants and open access users of a Web-based physical activity intervention. The most important findings were differences in adherence, attrition, and repeated participation between trial participants and open access users. Furthermore, reminder emails had a differential effect on attrition in trial participants and open access users. Assessing the data over time, there was an increase in the proportion of women using Active-online but no consistent trends in terms of adherence.

The yearly number of recorded visits on Active-online decreased from over 40,000 in 2003-2004 to less than 9,000 in 2008-2009. The most likely reason for this decrease was a decline in promotional efforts because there has been no active promotion of the website since 2008. Despite the decrease in the absolute number of visits, it is encouraging that even without active promotional strategies, Active-online still yielded around 23 visits per day in 2008-2009. Furthermore, no consistent time trends in adherence and patterns of individual intervention use were observed in open access users between 2003 and 2009.

Different reasons may be responsible for the increase in the proportion of women using Active-online between 2003 and 2009. For one thing, the proportion of women using the Internet has increased steadily in Switzerland from 23% in 1997 to 44% in 2006 [28]. Moreover, women are generally more interested in health information and use a wider spectrum of information sources [29]. Specifically, "online" women are more likely to use the Internet to look for health information than "online" men [30,31].

Trial participants differed in several ways from open access users. Adherence of trial participants during the first visit was generally lower. Only the small proportion that became involved with the intervention spent as much or more time in the tailored modules compared with open access users. The additional baseline data assessment in the trial context is a likely reason for the low use during the first visit in trial participants. However, comparing trial participants and participants of the feasibility study [23], in the latter group adherence was higher and more similar to the patterns observed in open access users (data not shown). Therefore, adherence may vary in different controlled study settings.

Trial participants were significantly more likely to visit the website repeatedly compared with open access users. Furthermore, when analyzing longest visits (Table 2), we found that adherence was similar or higher in trial participants compared with all open access users. However, registered open access users still showed higher adherence during their longest visit. In a study comparing public registrants of a cognitive behavior therapy website with trial participants, the latter were more likely to adhere to the full treatment program [17,32]. In that study, trial participants were contacted weekly by phone, suggesting that the formal structure of the trial and the personal contacts may have increased compliance in trial participants compared with public registrants [17,32].

In the open access context, registration did not achieve high levels of repeated participation (Figure 4 and Figure 5). This indicates that reminder emails (with the same content) may not have the same effect in different contexts. Having agreed to participate in a study, trial participants may have felt more committed to react to reminder emails. We did find, however, that adherence during the first visit was significantly higher among registered open access users compared with unregistered users (Table 2). For example, there was a large difference in visit duration between registered open access users and all open access users. The differences in adherence were supported by the attrition curves for the duration of the first and longest visits (Figure 6 and Figure 7). The registration process itself is very brief and cannot explain the large difference in visit duration. Therefore, registered open access users seem to have been more motivated to use the intervention thoroughly compared with unregistered users.

Open access users and trial participants who started a tailored module spent an average of 7.5 and 9.2 minutes in the modules, respectively. Other studies have found similar visit durations, for example, an average of 9 minutes was found among participants of a randomized study regarding another physical activity website [9], an average of 7.1 minutes per visit was found on a tailored physical activity Internet intervention in a randomized study setting [11], and an average of 7 minutes was found for visitors of a smoking cessation website [33].

Only about 2% of the open access visits on Active-online between 2003 and 2009 were repeated visits (Table 1). A Web-based behavior change program for healthy body weight and healthy lifestyle, in which an email reminder strategy similar to ours was used, resulted in about 10% of users visiting the website more than once [20]. However, registration was compulsory for users, making it easier for the study investigators to detect repeated visits and possibly resulting in selection by more motivated users. Two smoking cessation websites yielded almost 20% [15] and 27% [16] of registered visitors returning to the website, respectively. When considering only registered users in our study, one quarter of the registered open access users and two thirds of the trial participants visited the intervention more than once.

In a previous study, the main predictors of repeated participation in a behavior change program for healthy body weight were older age, never having smoked, meeting the guidelines for moderate physical activity and vegetable consumption, and being obese [20]. In our study, older age was confirmed as a predictor of repeated participation, but smoking, BMI, and meeting HEPA recommendations were not. We did not obtain information about vegetable consumption. The only other significant predictors documented in our study were male sex among open access users (which was not significant in trial participants) and Swiss nationality, which achieved borderline significance in trial participants only. There was a tendency for more highly educated trial participants to have been more likely to return, an effect that was also reported for an online smoking cessation program [15]. In another study, repeated use of an interactive coaching program for smoking cessation was predicted by female sex and older age, among other smoking-related variables [16].

Nonusage attrition was much higher for open access users (all open access users as well as the subgroup of registered open access users only) than for trial participants. Similar, although less pronounced, results have been reported for spontaneous users of a cognitive behavior therapy website compared with participants in an RCT through the same website [17]. Attrition curves of open access visitors to other websites in the domains of cognitive behavior therapy [18] and the promotion of sensible drinking [19] were also comparable (see Ware et al [14], Figure 2, which shows different published attrition curves).

### Limitations

The open structure of Active-online has advantages regarding dissemination and use of the intervention in that visitors are free to switch between modules, to open several windows concurrently, and to use the tailored intervention without registering. Thus, more individuals may be willing to participate in the intervention. However, this open structure also has some limitations. For example, a repeated visit of an unregistered user is recorded as a new first visit. Furthermore, an individual may open more than one tailored intervention browser window resulting in multiple new visits being recorded in the database. Individuals are also free to stop the intervention at any point, which can produce large amounts of missing data if the intervention is terminated before all questionnaires are completed. Nevertheless, our study results provide insight into an open access Web-based physical activity intervention delivered under real-world conditions and allow comparisons of use and users over time and in different contexts.

Another limitation is the lack of information on the sociodemographic background of open access users and additional potential predictors of repeated website usage. During the development of Active-online it was decided not to include questions ascertaining sociodemographic variables (with the exception of sex and age) at the start of the tailored intervention. There was a concern that this may discourage entering the intervention for individuals who may be unwilling to reveal personal information or to spend time completing questions not related to tailored feedback. Ideally, a newer version of Active-online may include a brief questionnaire with questions related to smoking, BMI, socioeconomic status, education, and nationality, for example. Finally, we compared open access users visiting Active-online between 2003 and 2009 with trial participants visiting the website between 2006 and 2007. Thus, potential period effects may have influenced the differences observed in our analyses. However, Table 2 does not suggest specific time trends in adherence to the intervention; therefore, we think it was justifiable to use the full time range of data collected for open access users.

### Conclusions

It is important to acknowledge that adherence, patterns of individual use, repeated participation, and attrition on a Web-based individually tailored physical activity intervention may differ between open access users and trial participants. Moreover, reminder emails to encourage repeated participation may not have the same effect in different contexts. These issues are important when interpreting and generalizing results of randomized controlled effectiveness trials.

## Abbreviations

BMI: body mass index
CI: confidence interval
HEPA: health-enhancing physical activity
IQR: interquartile range
RCT: randomized controlled trial
